# Knockdown of LncRNA CCAT1 Attenuates ox‐LDL‐Induced Inflammation in THP1‐Derived Macrophages via the miR‐296‐3p/FOSL1 Axis

**DOI:** 10.1155/cdr/9277233

**Published:** 2025-12-22

**Authors:** Haiping Zhang, Feng Liu, Guihua Miao, Yijiao Wang, Lijun Zhang, Chun Yang, Kai Huang, Fengyun Guo, JunBo An, Binfeng He, Jinshan Ye

**Affiliations:** ^1^ Department of Cardiology, Kunming Tongren Hospital, Kunming, Yunnan, China; ^2^ Department of Cardiology, Jintang County First People′s Hospital, Chengdu, Sichuan, China; ^3^ Department of General Practice, Xinqiao Hospital, Chongqing, China, xqhospital.com.cn

**Keywords:** atherosclerosis, CCAT1, FOSL1, macrophage, miR-296-3p

## Abstract

Macrophage‐driven inflammation, induced by dysfunctional lipid metabolism, is a pivotal process in the pathogenesis of atherosclerosis (AS). While long noncoding RNAs (LncRNAs) are established regulators, the specific mechanisms governing their roles remain largely uncharacterized. Here, we demonstrate that the LncRNA colon cancer–associated transcript 1 (CCAT1) is significantly upregulated in THP1‐derived macrophages upon stimulation with oxidized low‐density lipoprotein (ox‐LDL). Silencing LncRNA CCAT1 markedly attenuated the ox‐LDL‐induced inflammatory response, establishing its proinflammatory function. Mechanistically, we identified miR‐296‐3p as a direct downstream target of LncRNA CCAT1, which acts as a molecular sponge. This interaction was validated by dual‐luciferase and RNA pull‐down assays. Furthermore, we show that miR‐296‐3p suppresses inflammation by directly targeting the mRNA of FOS‐like antigen 1 (FOSL1). Crucially, overexpression of LncRNA CCAT1 increased FOSL1 levels, confirming this regulatory axis. Collectively, our findings delineate a novel pro‐inflammatory pathway where LncRNA CCAT1 promotes macrophage inflammation by sponging miR‐296‐3p, thereby derepressing its target FOSL1. Therefore, targeting LncRNA CCAT1 represents a promising therapeutic strategy for treating atherosclerotic cardiovascular disease.

## 1. Introduction

AS is a prominent cardiovascular disease (CVD) and a leading cause of vascular‐related mortality worldwide [1]. Chronic inflammation is a hallmark of AS [2], with macrophages playing a crucial role in driving the inflammatory response at every stage of the disease [3–5]. The macrophage‐mediated inflammatory response in AS encompasses several key steps, including macrophage recruitment through endothelial activation, phagocytosis of modified lipoproteins, secondary necrosis of cells, and the subsequent release of numerous proinflammatory mediators. Additionally, abnormal macrophage death processes such as pyroptosis, necroptosis, necrosis, and ferroptosis contribute to exacerbating vascular inflammation, necrotic core formation, and plaque development [6, 7]. Emerging studies highlight novel mechanisms underlying the macrophage‐mediated inflammatory response in dyslipidemic conditions, such as NLRP3 inflammasome activation, autophagy dysfunction, and proinflammatory signaling pathways mediated by TLRs [2, 8]. However, the precise mechanisms remain to be fully elucidated.

Epigenetic modifications, encompassing DNA/RNA methylation, histone modifications, and noncoding RNA, represent critical and reversible regulatory mechanisms of gene expression, independent of altering DNA sequences, and are triggered by environmental stress, pathological stimuli, among other factors [9, 10]. Mounting evidence suggests the involvement of epigenetic modifications in gene expression during the initiation and progression of atherosclerosis (AS), thus labeling AS as an epigenetic disease [11, 12]. Recent studies have illuminated the vital role of noncoding RNAs, such as miRNAs and LncRNAs, in modulating the macrophage inflammatory response in AS development [12]. Several studies have identified various LncRNAs, including H19 [13], TUG1 [14], and HOTAIR [15], as regulators of macrophage inflammatory responses involved in AS progress.

Recently, numerous reports have highlighted colon cancer–associated transcript 1 (CCAT1), a LncRNA, as a novel inflammatory regulator in various diseases; however, its exact role in the inflammatory response remains controversial. Chen and colleagues discovered that cigarette smoke extract (CSE) induced LncRNA CCAT1 expression in human bronchial epithelial (HBE) cells, and silencing LncRNA CCAT1 significantly suppressed CSE‐induced production of inflammatory mediators through the upregulation of miR‐152‐3p [16]. Other studies have reported that LPS increased LncRNA CCAT1 expression, and pharmacological downregulation of LncRNA CCAT1 attenuated LPS‐induced inflammation in skin keratinocyte HaCaT cells [16]. Conversely, in kidney epithelial HK2 cells, LPS reduced LncRNA CCAT1 expression, and LncRNA CCAT1 demonstrated an anti‐inflammatory role by acting as a sponge for miR‐155 [17]. Recent investigations have shown high expression of LncRNA CCAT1 in the M1 phenotype of tumor‐associated macrophages (TAMs), with downregulation of LncRNA CCAT1 promoting a phenotype shift from M1 to M2 [18]. Nevertheless, the specific role of LncRNA CCAT1 in macrophage inflammatory responses in AS progression remains unclear.

Herein, we investigated the role of LncRNA CCAT1 in macrophage inflammatory responses to dyslipidemia. Our findings establish LncRNA CCAT1 as a key proinflammatory regulator in macrophages exposed to ox‐LDL. We demonstrate that it functions by sponging miR‐296, thereby enhancing the expression of FOS‐like antigen 1 (FOSL1).

## 2. Materials and Method

### 2.1. Reagents and Cell line

The completed culture medium of THP1 (#CH1137‐5) was from Newgainbio Inc (Wuxi, China); DMEM (#C11995500BT), 0.25% Trysin‐EDTA (#25200‐072), and Opti‐MEM reduced serum medium (#31985062) were obtained from Thermo Fisher Scientific Inc (Shanghai, China). ox‐LDL (#YB‐002) was from YiYuan Bio Inc (Guangzhou, China). RNAiso Plus (TRIzol) reagents (#9108Q) and Prime Script^RT^ reagent Kit (#RR037A) were purchased from Takara Biomedical Technology Co., Ltd. (Beijing, China). The miRNA First‐Strand cDNA Synthesis (Tailing Reaction) kit (#B532451), SanPrep Column microRNA Extraction Kit (#B518811), RNA‐Protein Pull Down Kit (#B605110‐0006), human ELISA kits for IL‐6 (#D711391), IL‐1*β* (#D711068), IL‐8/CXCL8 (#D711366), and IL‐10 (#D711393) were purchased from Sangon Biotech. RIPA Lysis Buffer (#P0013B), protease inhibitor cocktail for general use (#P1005), and Lipo8000 Transfection Reagent (#C0533) were from Beyotime Biotechnology (Shanghai, China). Fetal bovine serum (FBS) (#40130ES76), Hieff qPCR SYBR Green Master Mix (#11201ES03), Dual Luciferase Reporter Gene Assay Kit (#11402ES60), Super ECL Detection Reagent ECL (#36208ES76) were purchased from Yeasen Inc (Shanghai, China). Advanced DNA RNA Transfection Reagent (#AD600025) was purchased from ZETA LIFE (San Francisco, United States).

The antibodies used were anti‐FOSL1 Antibody (CST, #5281), anti‐NLRP3 Antibody (CST, # 15101), anti‐Phospho‐NF‐*κ*B p65 (Ser536) (p‐p65) (CST, #3033), anti‐NF‐*κ*B p65 (p65) (CST, #8242), recombinant anti‐GAPDH antibody (Servicebio, #GB11002), and HRP‐conjugated goat anti‐rabbit IgG (H + L) (Servicebio, #GB23303).

THP1 (#SCSP‐567) and 293T (#SCSP‐502) were obtained from Cell Bank/Stem Cell Bank, CAS (Shanghai, China). THP1 cells were cultured with THP1 completed medium. To induce THP1‐derived macrophages, the THP1 cells were treated with 100 ng/ml phorbol 12‐myristate 13‐acetate (PMA) for 24 h. 293T were cultured in DMEM supplemented with 10% FBS.

### 2.2. RNA Extraction and Real‐Time qPCR

Total RNA from the treated THP1 cells and THP1‐driven macrophages was isolated using the RNAiso Plus reagent following the manufacturer′s protocol. The miRNA from cell samples was isolated using the SanPrep Column microRNA Extraction Kit. Subsequently, the total RNA was reverse‐transcribed into cDNA using the Prime Script RT reagent Kit or miRNA First Strand cDNA Synthesis Kit (Tailing Reaction). The relative expression of the target gene was detected using Hieff qPCR SYBR Green Master Mix on the BIO‐RAD CFX96 platform, employing the 2^-△△Ct^ method. GAPDH or U6 was used as the normalization control. The details of the primers are provided in Table S1.

### 2.3. DNA and RNA Transfections

The LncRNA CCAT1 siRNA, FOSL1 siRNA, miR‐296‐3p mimic, miR‐296‐3p inhibitor, and LncRNA CCAT1 overexpression (LncRNA CCAT1‐OE) plasmid was custom designed and synthesized by Sangon Biotech. The primer sequences were as follows: LncRNA CCAT1 siRNA (siLncRNA CCAT1)#1: 5 ^′^‐GCUGGAUGAAUGUUUAACUTT‐3 ^′^, CCAT1 siRNA#2: 5 ^′^‐GACCAUAAGAAGAUCAUAUTT‐3 ^′^, CCAT1 siRNA#3: 5 ^′^‐GGAGGGUGCUUGACAAUAATT‐3 ^′^, FOSL1 siRNA#1: 5 ^′^‐ GGAGCUGCAGUGGAUGGUATT‐3 ^′^, FOSL1 siRNA#2: 5 ^′^‐ GCAGGCGGAGACUGACAAATT‐3 ^′^, FOSL1 siRNA#3: 5 ^′^‐ CCAGCCUGGUCUUCACCUATT‐3 ^′^. A negative control (NC) sequence was also synthesized: 5 ^′^‐ACGUGACACGUUCGGAGAATT‐3 ^′^, miR‐296‐3p mimic: 5 ^′^‐ GAG GGUUGGGUGGAGGCUCUCC‐3 ^′^, miR‐296‐3p inhibitor: 5 ^′^‐GGAGAGCCUCCACCCAACCCUC‐3 ^′^, miRNA‐NC: 5 ^′^‐CAGUACUUUUGUGUAGUACAA‐3 ^′^.

THP1 cells were centrifuged and resuspended in Opti‐MEM medium before being seeded into a 6‐well plate. RNA oligos at a concentration of 100 nM or LncRNA CCAT1‐OE plasmid were mixed with Advanced DNA RNA Transfection Reagent and incubated for 15 min at RT. The resulting mixture was then transfected into THP1 cells and incubated for 24 h.

### 2.4. Dual‐Luciferase Assay

The interaction sites between LncRNA CCAT1 and miR‐296‐3p, as well as FOSL1 and miR‐296‐3p, were predicted using the StarBase database (http://starbase.sysu.edu.cn/) and miRTarBase (https://mirtarbase.cuhk.edu.cn/).

The binding site sequences of LncRNA CCAT1 and FOSL1 3 ^′^ UTR region were synthesized and cloned into the pmirGLO luciferase plasmid by Sangon Biotech. For assessing the interaction of LncRNA CCAT1 with miR‐296‐3p, 293T cells were seeded in a 24‐well plate overnight. The pmirGLO‐CCAT1 wild type (CCAT1 WT) or pmirGLO‐CCAT1 mutation (CCAT1 Mut), along with miR‐296‐3p mimic, were co‐transfected into the 293T cells using Lipo8000 for 24 h. Similarly, to evaluate the binding of FOSL1 3 ^′^ UTR with miR‐296‐3p, pmirGLO‐FOSL1 wild type (FOSL1 WT) or‐FOSL1 mutation (FOSL1 Mut), and miR‐296‐3p mimic were cotransfected into 293T cells. Dual‐luciferase assays were conducted using the Dual Luciferase Reporter Assay System.

### 2.5. RNA Pull‐Down Assay

The biotinylated (bio)‐miR‐296‐3p mimic (WT), bio‐miR‐296‐3p mutation mimic (Mut), or biotinylated NC was designed and synthesized by Sangon Biotech. The RNA pull‐down assay was conducted following the manufacturer′s instructions. Briefly, THP1‐derived macrophages were stimulated with 50 *μ*g/mL of ox‐LDL for 24 h, and the cell lysates were prepared using an IP lysis buffer. These lysates were then incubated with the (bio)‐miR‐296‐3p mimic (WT), bio‐miR‐296‐3p Mut, or biotinylated NC–loaded streptavidin magnetic beads in 50% glycerol overnight. Subsequently, the RNA‐bead complexes were washed three times, and RNAiso Plus reagent was utilized to extract the bound RNA. The expression levels of LncRNA CCAT1 were assessed through qPCR.

### 2.6. Western Blot

The total cellular proteins were lysed using RIPA lysis buffer. Subsequently, these protein samples were separated on an SDS‐PAGE gel and transferred to PVDF membranes. The membranes were blocked with 5% nonfat milk at room temperature for 2 h and then incubated with the target protein antibody overnight at 4°C. A secondary antibody was used to incubate the membranes for 1 h at room temperature, and the membranes were visualized using the Amersham Imager 600 system and ChemiDoc TM MP imaging system (BIO‐RAD, United States).

### 2.7. ELISA

To evaluate the inflammatory conditions following the exposure of THP1‐derived macrophages to ox‐LDL, the levels of IL‐1*β*, IL‐6, IL‐8, and IL‐10 in the culture medium were measured using an ELISA kit according to the manufacturer′s instructions.

### 2.8. Statistical Analysis

All experiments were performed with at least three replicates. Statistical analysis was performed using GraphPad Prism 9 (Version 9.0.0, GraphPad Software, United States). Before making statistical comparisons, the assumptions for parametric tests were rigorously evaluated. The normality of data distribution within each group was assessed using the Shapiro–Wilk test. Homoscedasticity (homogeneity of variances) across groups was confirmed using Levene′s test. A significance level of *α* = 0.05 was established for these diagnostic tests. If the data met both normality and homoscedasticity assumptions, group comparisons were conducted using Student′s *t*‐tests for comparisons between two groups, and one‐way ANOVA followed by Tukey′s post hoc test for comparisons among three or more groups. If either the normality or homoscedasticity assumption was violated (*p* < 0.05), appropriate nonparametric alternatives were employed using the Mann–Whitney *U* test for two‐group comparisons and the Kruskal–Wallis test followed by Dunn′s post hoc test for multiple comparisons among three or more groups. Statistically significant differences were defined as *p* < 0.05.

## 3. Result

### 3.1. ox‐LDL‐Induced LncRNA CCAT1 Expression in THP1‐Driven Macrophages

To investigate the role of LncRNA CCAT1 in the ox‐LDL‐induced inflammatory response in macrophages, we first assessed the LncRNA CCAT1 expression following ox‐LDL exposure in THP1‐derived macrophages. In Figure 1a, it is evident that the expression of LncRNA CCAT1 increases significantly when THP1‐derived macrophages are incubated with 10, 20, and 50 *μ*g/mL of ox‐LDL for 24 h compared to the control group (*p* < 0.05). Moreover, the expression of LncRNA CCAT1 shows a dose‐dependent manner. Additionally, the levels of the inflammatory cytokines IL‐6, IL‐8, IL‐1*β*, and IL‐10 in culture medium are upregulated upon THP1‐derived macrophage exposure to ox‐LDL (Figures 1b, 1c, 1d, and 1e). THP1‐derived macrophages treated with 50 *μ*g/mL of ox‐LDL for 6, 12, and 24 h exhibit higher LncRNA CCAT1 expression than the control group (*p* < 0.05) (Figure 1f). Furthermore, the levels of IL‐6, IL‐8, IL‐1*β*, and IL‐10 in culture medium are also elevated compared to the control group, with the highest levels observed in the 50 *μ*g/mL group (Figure 1g, 1h, 1i, and 1j). These findings suggest that ox‐LDL‐induced LncRNA CCAT1 expression, and it might involve in the inflammatory response triggered by ox‐LDL in THP1‐derived macrophages.

Figure 1ox‐LDL‐induced LncRNA CCAT1 expression and inflammatory response. THP1‐derived macrophages were incubated with indicated doses ox‐LDL for 24 h. (a) qPCR detected the expression of LncRNA CCAT1. (b–e) ELISA method evaluated the levels of IL‐8, IL‐6, IL‐1*β*, and IL‐10 in culture medium. THP1‐derived macrophages were incubated with indicated 50 *μ*g/mL ox‐LDL for indicated time points. (f) qPCR measured LncRNA CCAT1 expression. (g–j) ELISA method detected the levels of IL‐8, IL‐6, IL‐1*β*, and IL‐10 in culture medium.  ^∗^ *p* < 0.05 by one‐way ANOVA followed by Tukey′s multiple comparisons test. Experiment was conducted with three replicates.

**Figure:**
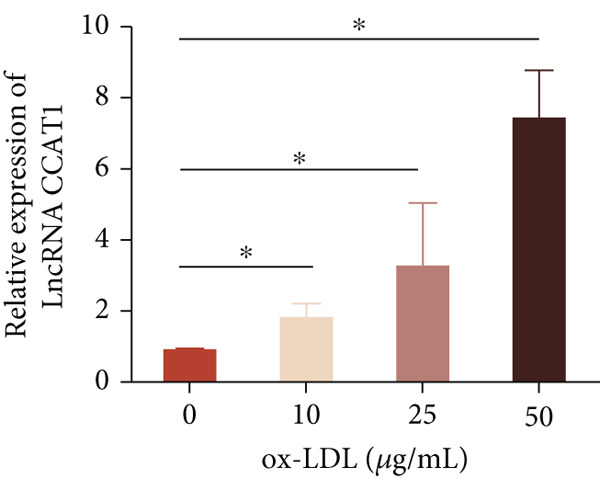
(a)

**Figure:**
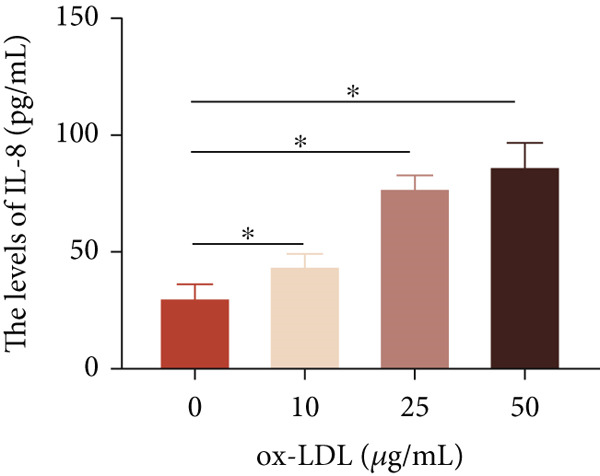
(b)

**Figure:**
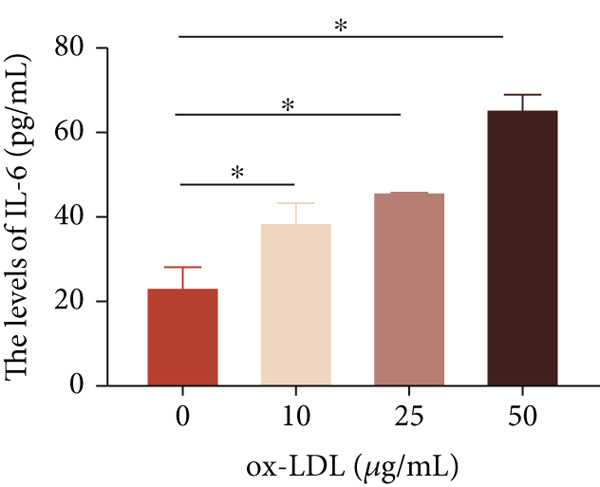
(c)

**Figure:**
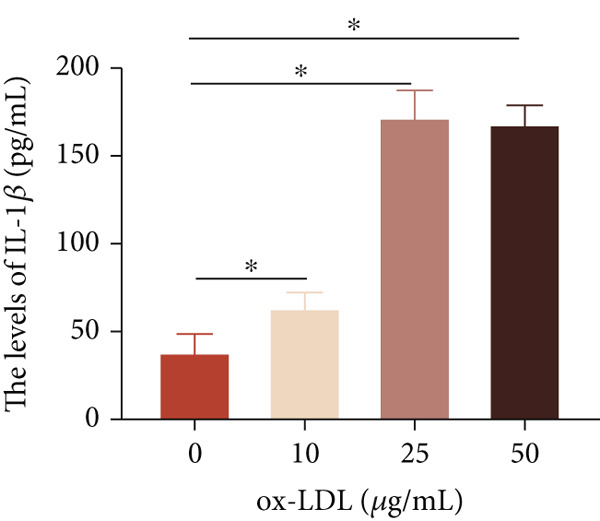
(d)

**Figure:**
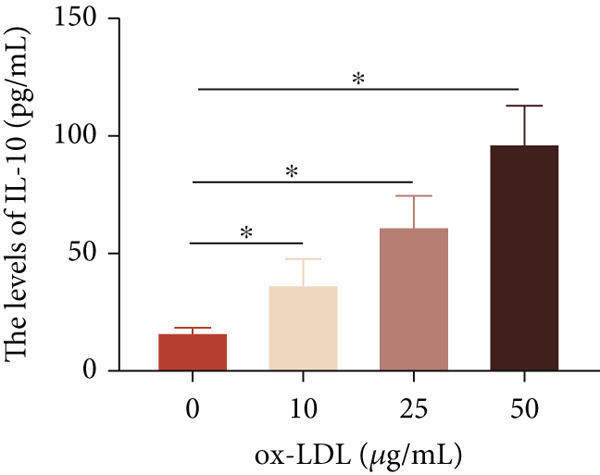
(e)

**Figure:**
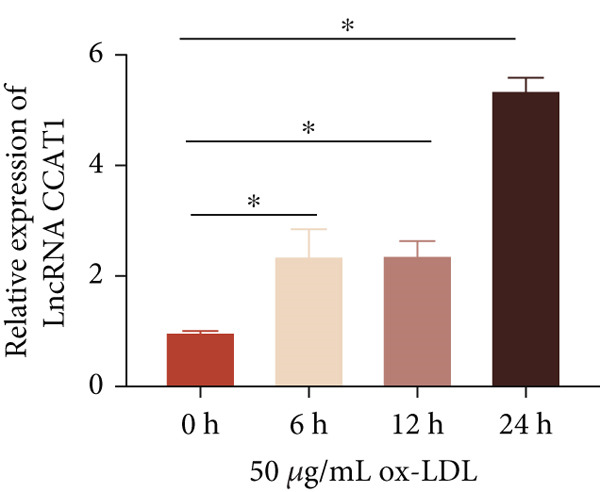
(f)

**Figure:**
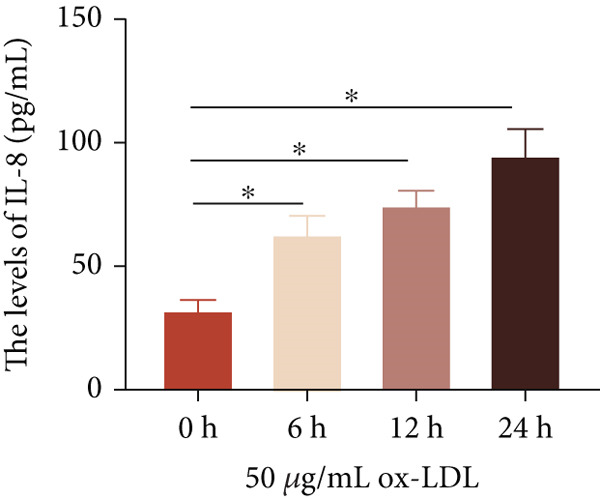
(g)

**Figure:**
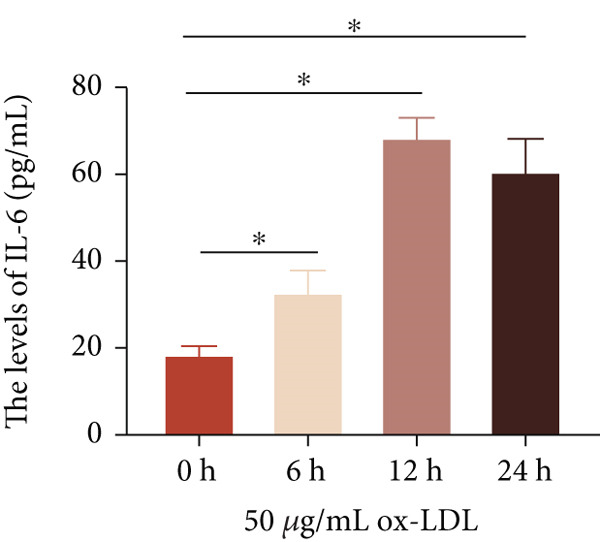
(h)

**Figure:**
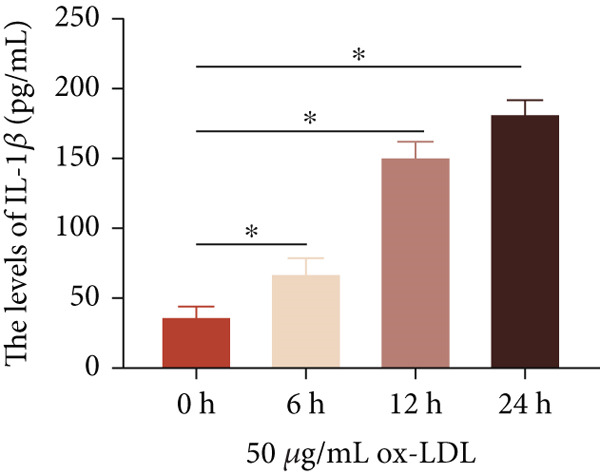
(i)

**Figure:**
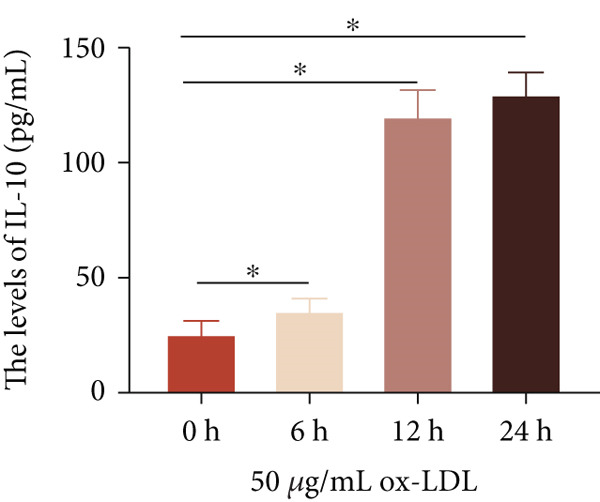
(j)

### 3.2. Silencing LncRNA CCAT1 Attenuated ox‐LDL‐Induced THP1‐Driven Macrophage Inflammatory Response

To confirm the role of LncRNA CCAT1 in ox‐LDL‐induced macrophage inflammatory response, LncRNA CCAT1 in THP1 cells was silenced using LncRNA CCAT1 siRNA (siLncRNA CCAT1). The data indicated that three siRNA oligos significantly downregulated LncRNA CCAT1 expression. Among these, siRNA #2 (S2) was the most effective in silencing LncRNA CCAT1 and was chosen for further studies (Figure 2a). Subsequently, we evaluated the impact of LncRNA CCAT1 knockdown on ox‐LDL‐induced THP1‐driven macrophage inflammatory response. The results demonstrated that silencing LncRNA CCAT1 partly inhibited ox‐LDL‐induced LncRNA CCAT1 expression (Figure 2b). Additionally, the levels of IL‐6, IL‐8, and IL‐1*β* were notably reduced, while IL‐10 levels were higher in the LncRNA CCAT1 siRNA/ox‐LDL group compared to the ox‐LDL group (*p* < 0.05) (Figures 2c, 2d, 2e, and 2f). These findings suggest that LncRNA CCAT1 promotes ox‐LDL‐induced inflammatory responses, while silencing LncRNA CCAT1 offers a novel strategy to attenuate these inflammatory reactions.

Figure 2Knocking down LncRNA CCAT1 suppressed ox‐LDL‐induced inflammatory response. (a) qPCR detected the expression of LncRNA CCAT1 after THP1 cells were transfected with NC and three siRNA oligos for 24 h, respectively. THP1 cells were transfected with CCAT1 siRNA for 24 h, followed by a 24‐h incubation with 100 ng/mL of PMA to induce THP1‐derived macrophage. Subsequently, the cells were treated with 50 *μ*g/mL of ox‐LDL for an additional 24 h. (b) qPCR detected the expression of LncRNA CCAT1. (c–e) ELISA method detected the levels of IL‐8, IL‐6, IL‐1*β*, and IL‐10 in culture medium. ^ns^ *p* ≥ 0.05 and  ^∗^ *p* < 0.05 by one‐way ANOVA followed by Tukey′s multiple comparisons test. Experiment was conducted with three replicates.

**Figure:**
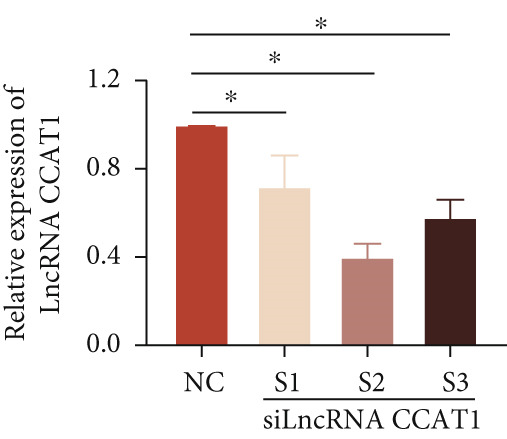
(a)

**Figure:**
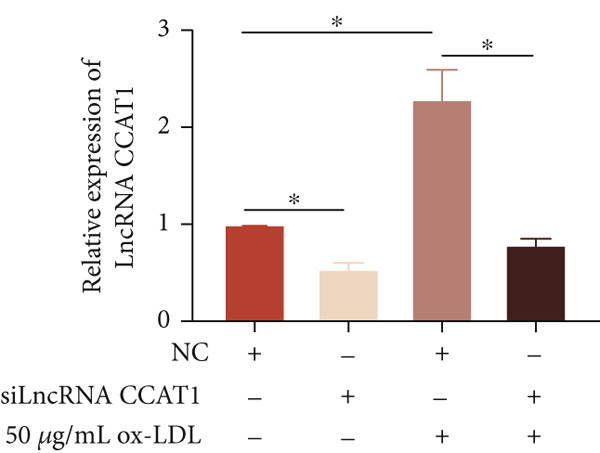
(b)

**Figure:**
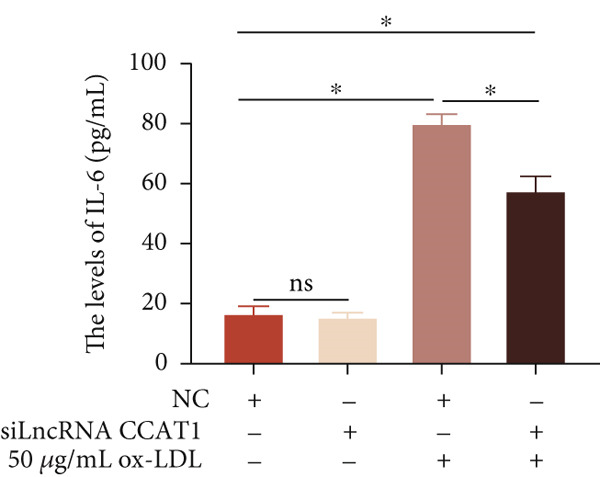
(c)

**Figure:**
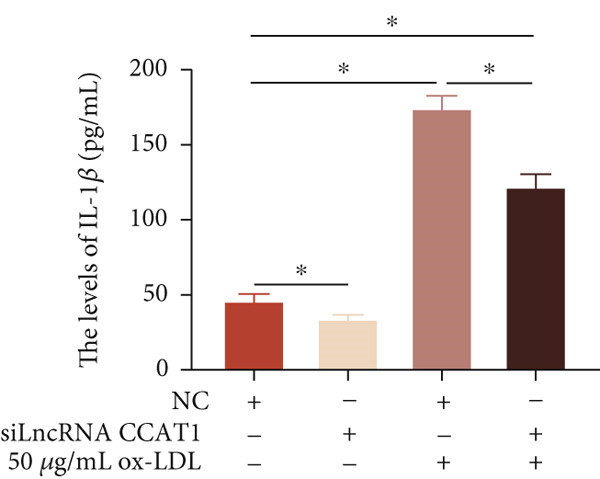
(d)

**Figure:**
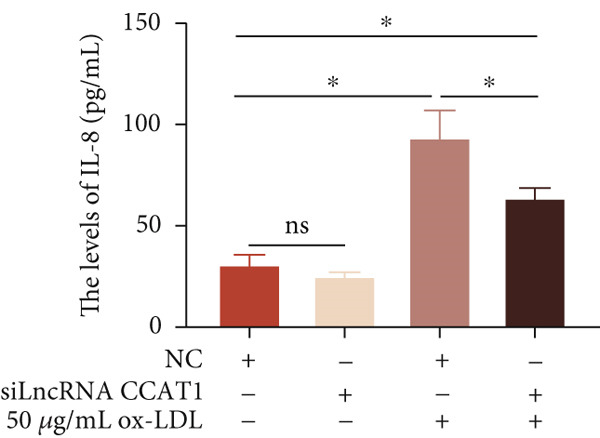
(e)

**Figure:**
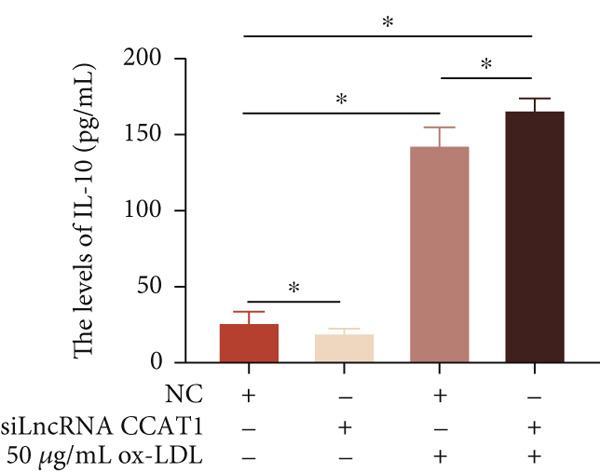
(f)

### 3.3. miR‐296‐3p Is as a Novel Target of LncRNA CCAT1

To investigate the novel mechanism of LncRNA CCAT1 regulation in ox‐LDL‐induced inflammatory response, we conducted target screening using StarBase and LncACTdb. Several genes were screened, including miR‐296‐3p, let‐7a, miR‐152‐3p, and miR‐454‐3p. Subsequently, LncRNA CCAT1 was knocked down in THP1 cells to further analyze the potential identified LncRNA CCAT1 targets. The results demonstrated a significant increase in miR‐296‐3p levels after treatment of THP1 cells with LncRNA CCAT1 siRNA (*p* < 0.05) (Figure 3a).

Next, we then evaluated the expression of miR‐296‐3p in THP1‐derived macrophages following ox‐LDL exposure. The data revealed that at the 24‐h time point, 20 and 50 *μ*g/mL of ox‐LDL suppressed miR‐296‐3p expression (Figure 3b), and its levels decreased after treating THP1‐derived macrophages with 50 *μ*g/mL of ox‐LDL for 12 and 24 h (Figure 3c). Moreover, the downregulation of LncRNA CCAT1 partially reversed the inhibitory effect of ox‐LDL on miR‐296‐3p (Figure 3d).

Figure 3miR‐296‐3P is sponged by LncRNA CCAT1. (a) qPCR detected the expression of LncRNA CCAT1, miR‐296‐3P, Let‐7a, and miR‐152‐5P after THP1 cells were transfected with LncRNA CCAT1 siRNA for 24 h, followed by treating 100 ng/mL PMA for another 24 h to induce THP1‐derived macrophage. ^ns^ *p* ≥ 0.05 and  ^∗^ *p* < 0.05 were determined using Student′s *t*‐test. Experiment was conducted with three replicates. (b) qPCR evaluated the expression of miR‐296‐3P after THP1‐derived macrophage was incubated with indicated doses ox‐LDL for 24 h. (c) The expression of miR‐296‐3P was evaluated by qPCR after THP1‐derived macrophage were incubated with 50 *μ*g/mL ox‐LDL for indicated time points. (d) The expression of miR‐296‐3P was evaluated by qPCR. THP1 cells were transfected with LncRNA CCAT1 siRNA for 24 h, followed by a 24‐h incubation with 100 ng/mL of PMA to induce THP1‐derived macrophage. Subsequently, the cells were treated with 50 *μ*g/mL of ox‐LDL for an additional 24 h. (e) Luciferase activity had been detected in 293T cells after these cells were transfected with pmirGLO‐CCAT1 WT or Mut and miR‐296‐3p mimics or NC mimics. ^ns^ *p* ≥ 0.05 and  ^∗^ *p* < 0.05 were determined using Student′s *t*‐test. Experiment was conducted with three replicates. (f) The enrichment levels of LncRNA CCAT1 in the bound RNA miR‐296‐3P had been examined by RNA pull‐down assay. ^ns^ *p* ≥ 0.05 and  ^∗^ *p* < 0.05 were determined using Student′s *t*‐test. Experiment was conducted with three replicates.

**Figure:**
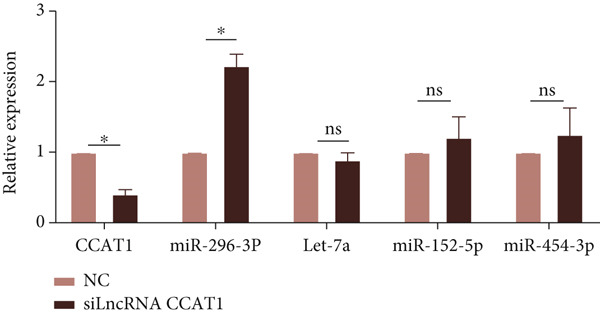
(a)

**Figure:**
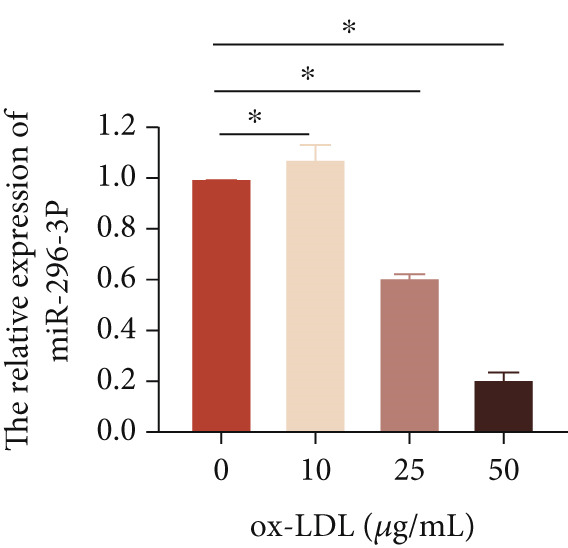
(b)

**Figure:**
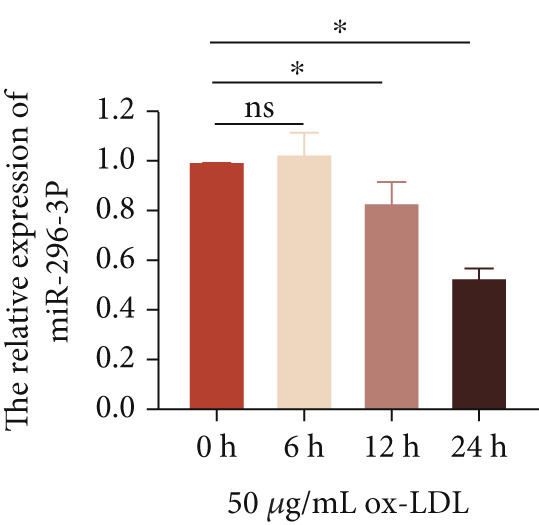
(c)

**Figure:**
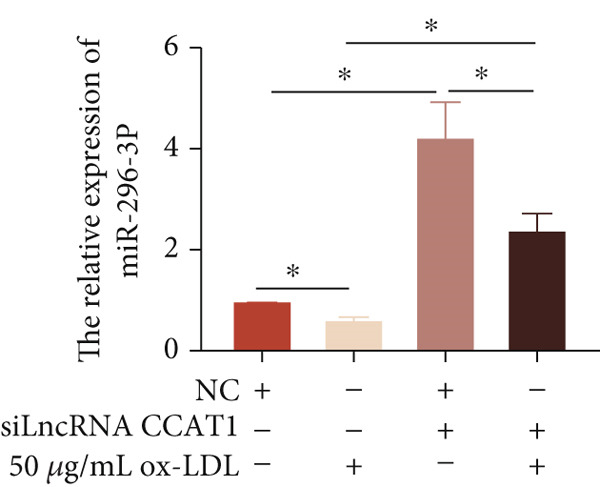
(d)

**Figure:**
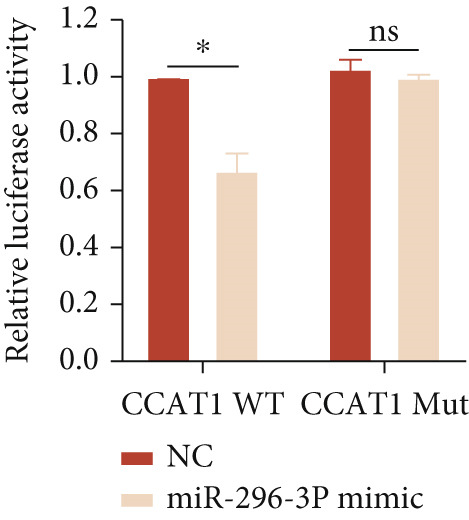
(e)

**Figure:**
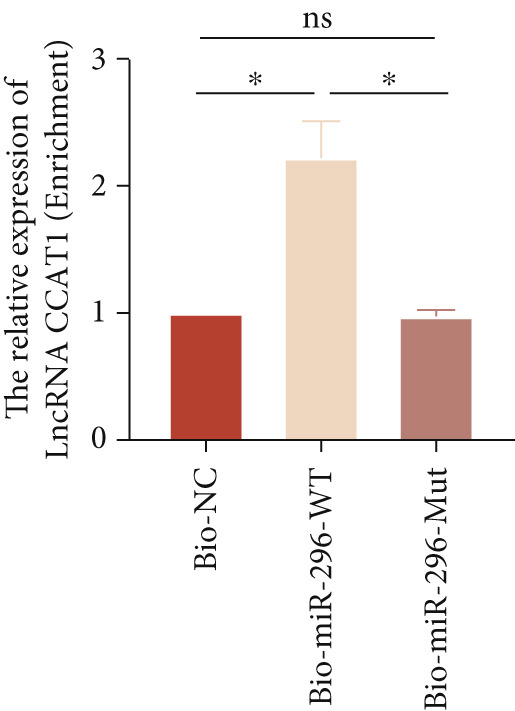
(f)

Furthermore, a luciferase assay was conducted to assess the binding of LncRNA CCAT1 to miR‐296 (Figure 3e). The results demonstrated the binding of LncRNA CCAT1 to miR‐296‐3p. Additionally, RNA pull‐down assay data indicated that miR‐396‐3p could interact with LncRNA CCAT1 (Figure 3f). This data suggests that LncRNA CCAT1 acts as a sponge for miR‐296‐3p. This data suggests that miR‐296‐3p is a novel target of LncRNA CCAT1.

### 3.4. miR‐296‐3p Suppressed ox‐LDL‐Induced THP1‐Driven Macrophage Inflammatory Response Through Targeting FOSL1

To investigate the role of miR‐296‐3p in the THP1‐driven macrophages inflammatory response induced by ox‐LDL, we upregulated the levels of miR‐296‐3p using a miR‐296‐3p mimic (Figure 4a) and assessed its impact on the expression of inflammatory cytokines. miR‐296‐3p reduced the levels of IL‐6, IL‐8, and IL‐1*β* in the absence or presence of ox‐LDL (Figures 4b, 4c, and 4d); however, IL‐10 levels did not show a significant decrease (Figure 4e). Moreover, miR‐296‐3p inhibiting with a miR‐296‐3p inhibitor strengthened ox‐LDL‐induced levels of IL‐6, IL‐8, and IL‐1*β* (Figures 4f, 4g, and 4h), while IL‐10 levels were decreased (Figure 4i). These findings demonstrate that miR‐296‐3p suppresses the ox‐LDL‐induced inflammatory response in THP1‐driven macrophages.

Figure 4miR‐296‐3P suppressed ox‐LDL‐induced THP1‐derived macrophage inflammatory. (a) The expression of miR‐296‐3P was detected by qPCR upon miR‐296‐3P mimic transfected into THP1 cells.  ^∗^ *p* < 0.05 were determined using Student′s *t*‐test. Experiment was conducted with three replicates. THP1 cells were transfected with miR‐296‐3P mimic for 24 h, followed by a 24‐h incubation with 100 ng/mL of PMA to induce THP1‐derived macrophage. Subsequently, the cells were treated with 50 *μ*g/mL of ox‐LDL for an additional 24 h. (b–e) The levels of IL‐6, IL‐8, IL‐1*β*, and IL‐10 of culture medium were detected by ELISA method. THP1 cells were transfected with miR‐296‐3P inhibitor for 24 h, followed by a 24‐h incubation with 100 ng/mL of PMA to induce THP1‐derived macrophage. Subsequently, the cells were treated with 50 *μ*g/mL of ox‐LDL for an additional 24 h. (f–i) ELISA method evaluated the levels of IL‐6, IL‐8, IL‐1*β*, and IL‐10 in culture medium. ^ns^ *p* ≥ 0.05 and  ^∗^ *p* < 0.05, by one‐way ANOVA followed by Tukey′s multiple comparisons test. Experiment was conducted with three replicates.

**Figure:**
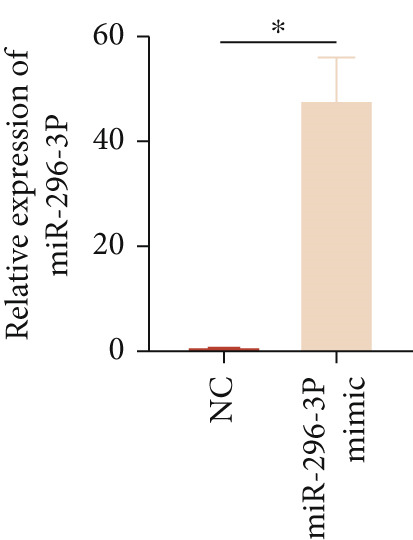
(a)

**Figure:**
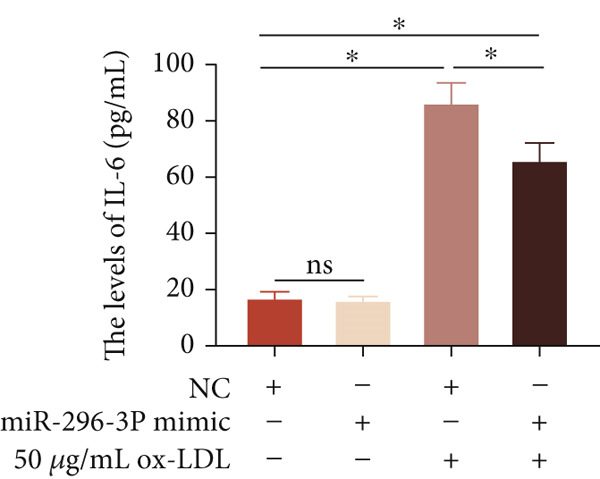
(b)

**Figure:**
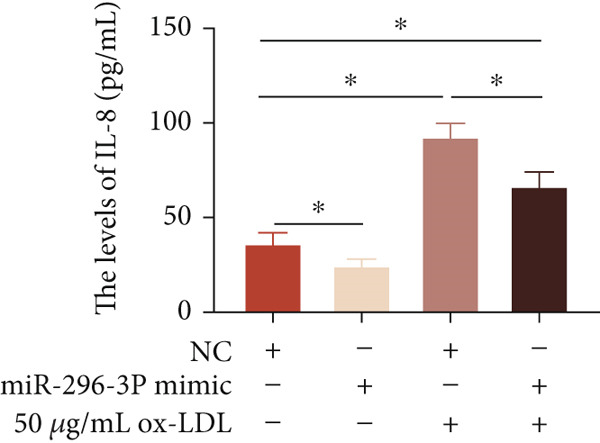
(c)

**Figure:**
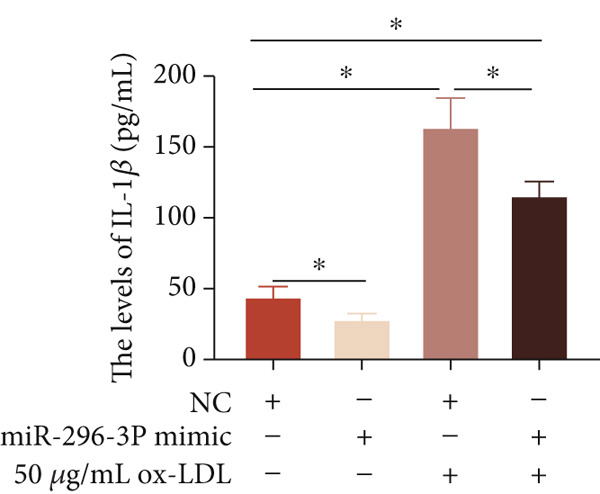
(d)

**Figure:**
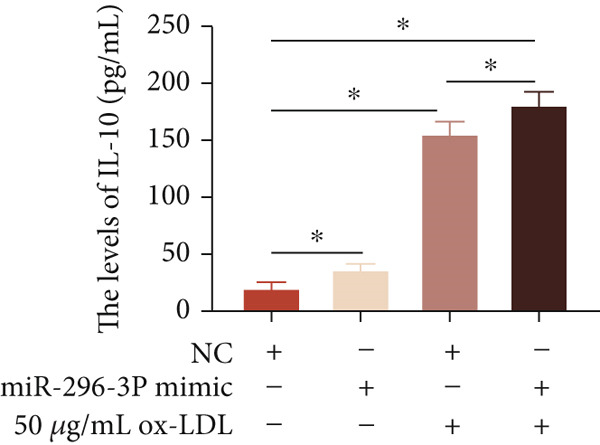
(e)

**Figure:**
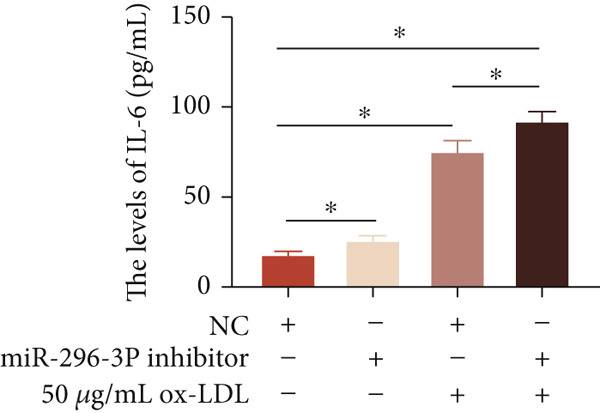
(f)

**Figure:**
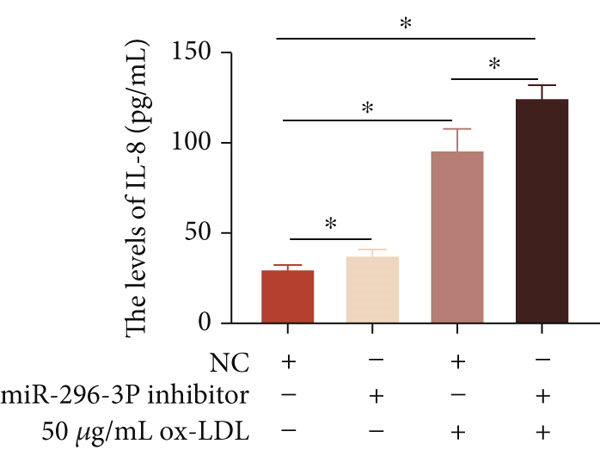
(g)

**Figure:**
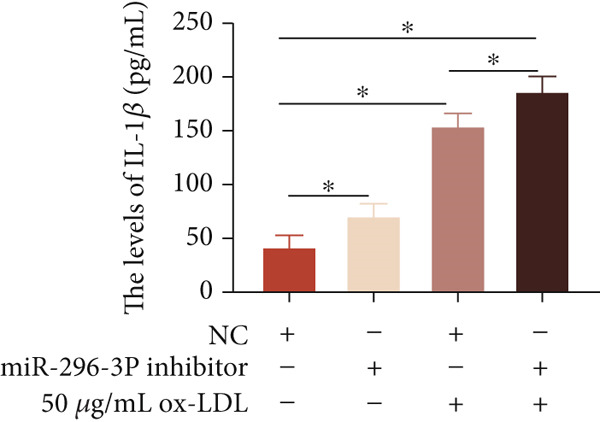
(h)

**Figure:**
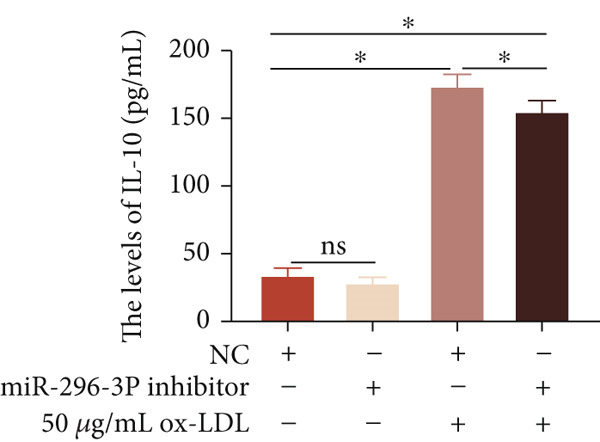
(i)

miRNAs play a crucial role in regulating cellular functions by binding to the 3 ^′^ UTR of target mRNAs [19]. The target mRNA FOSL1 was identified through miRTarBase and TarBase databases (Figure 5a). Subsequent investigations confirmed that miR‐296‐3p binds to the 3 ^′^ UTR of FOSL1 mRNA as demonstrated by dual‐luciferase assay (Figure 5b). Furthermore, miR‐296‐3p suppresses the expression of FOSL1 mRNA (Figure 5c,d). Conversely, inhibiting miR‐296‐3p increases the levels of FOSL1 (Figure 5e,f), indicating that FOSL1 is a target of miR‐296‐3p. Moreover, we observed that the upregulation of LncRNA CCAT1 led to elevated mRNA and protein levels of FOSL1 (Figure 5g,h). Additionally, LncRNA CCAT1 overexpression partially reversed the inhibitory effect of miR‐296‐3p on FOSL1 (Figure 5i,j), suggesting that LncRNA CCAT1 may facilitate FOSL1 expression by acting as a sponge for miR‐296.

Figure 5FOSL1 is target of miR‐296‐3P. (a) Venny confirmed that FOSL1 is a target of miR‐296‐3P by referencing the miRTarBase and TarBase databases. (b) Luciferase activity had been detected in 293T cells after these cells were transfected with pmirGLO‐FOSL1 WT or FOSL1 MUT and miR‐296‐3p mimics or NC mimics. The miR‐296‐3p mimic was transfected into THP1 cells for 24 h, followed by a 24‐h incubation with 100 ng/mL of PMA to induce THP1‐derived macrophage. (c, d) qPCR and Western blot detected the expression of FOSL1 mRNA and protein. miR‐296‐3p inhibitor transfected into THP1 cells for 24 h, followed by a 24‐h incubation with 100 ng/mL of PMA to induce THP1‐derived macrophage. (e, f) qPCR and Western blot detected the expression of FOSL1 mRNA and protein.  ^∗^ *p* < 0.05 was determined using Student′s *t*‐test. THP1 cells were transfected with LncRNA CCAT1‐OE plasmid for indicated time points and then incubated with 100 ng/mL of PMA for 24 h to induce THP1‐derived macrophage. (g, h) qPCR and Western blot detected the expression of FOSL1 mRNA and protein. THP1 cells were cotransfected with LncRNA CCAT1‐OE plasmid and miR‐296‐3P mimic for 24 h, incubated with 100 ng/mL of PMA for 24 h to induce THP1‐derived macrophage. (i, j) The level of FOSL1 mRNA and protein was detected by qPCR and western blot. ^ns^ *p* ≥ 0.05 and  ^∗^ *p* < 0.05 by one‐way ANOVA followed by Tukey′s multiple comparisons test. Experiment was conducted with three replicates.

**Figure:**
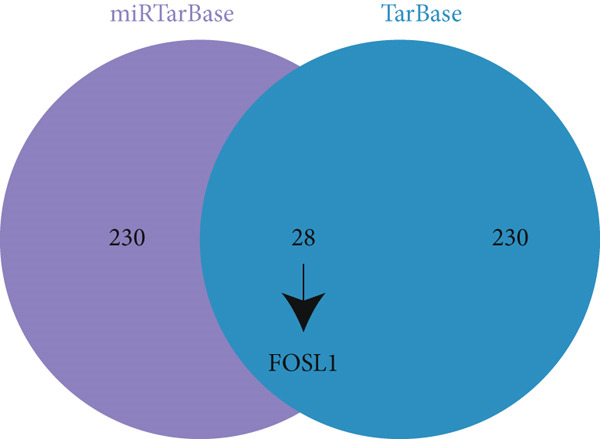
(a)

**Figure:**
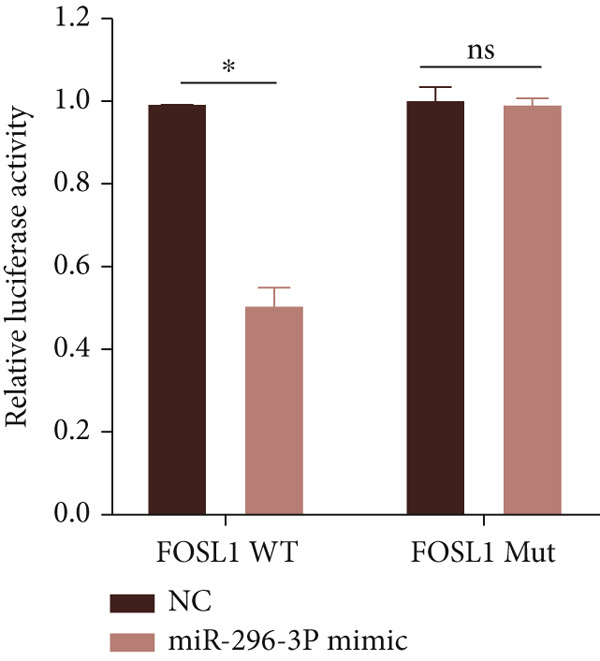
(b)

**Figure:**
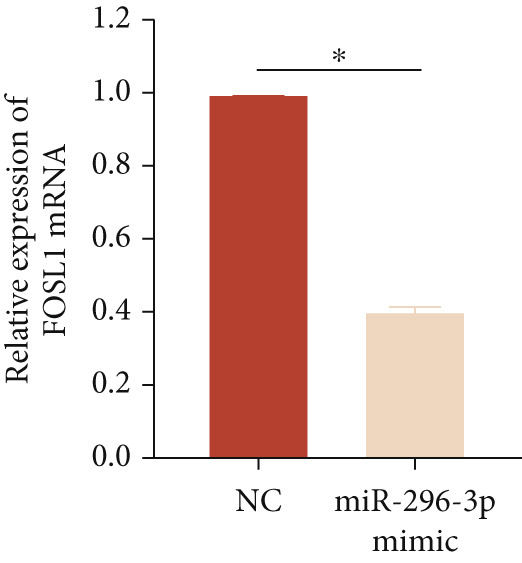
(c)

**Figure:**
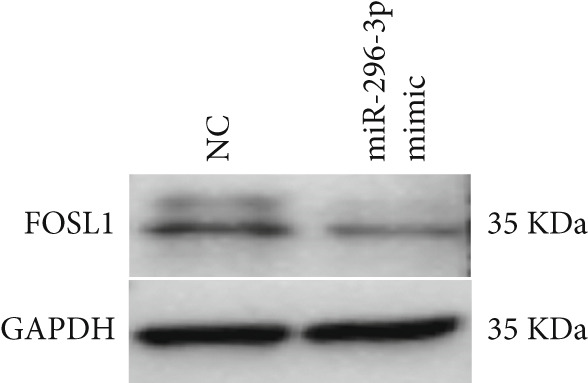
(d)

**Figure:**
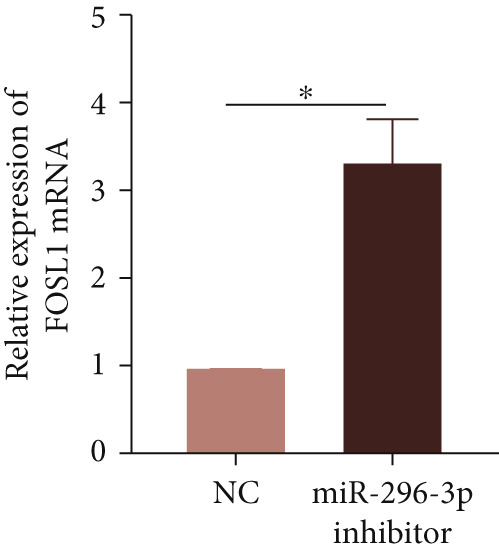
(e)

**Figure:**
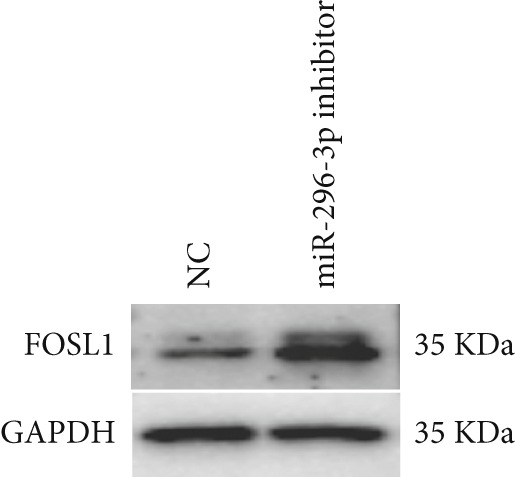
(f)

**Figure:**
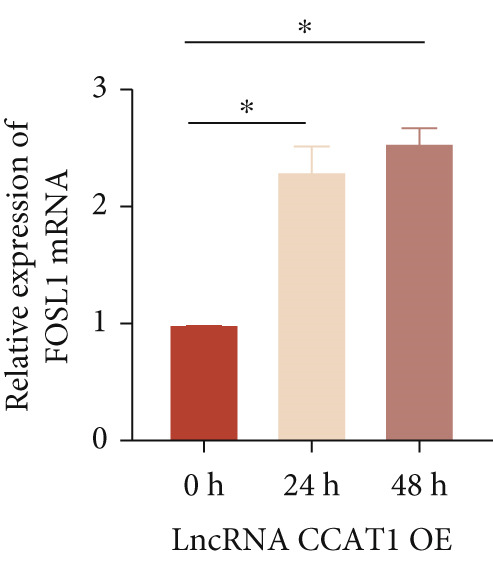
(g)

**Figure:**
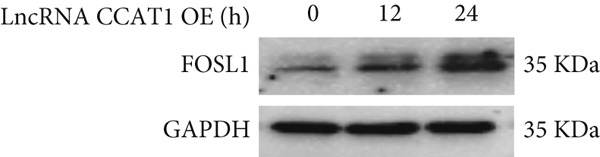
(h)

**Figure:**
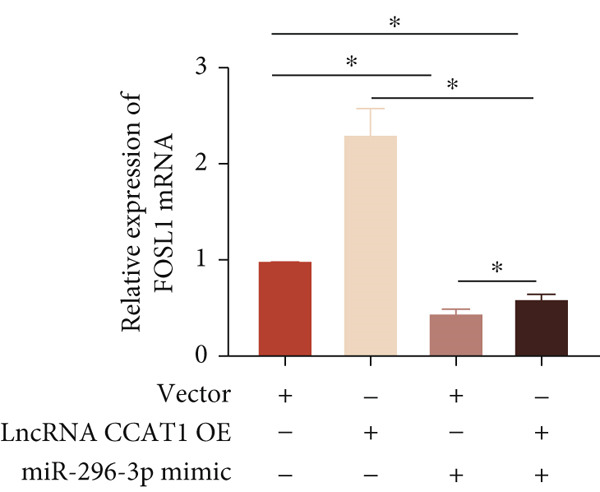
(i)

**Figure:**
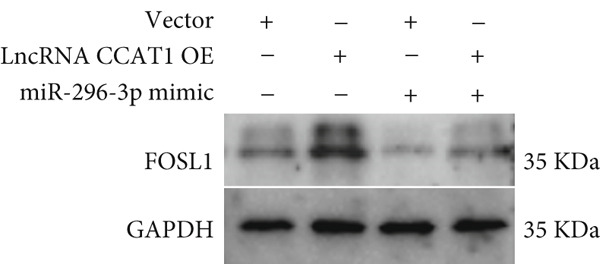
(j)

We further investigated the role of FOSL1 in regulating the inflammatory response. The levels of FOSL1 significantly increased when THP1‐derived macrophages were exposed to ox‐LDL, as depicted in Figure 6a,b. Figure 6c,d illustrated that three FOSL1 siRNA oligos markedly downregulated FOSL1 expression, with siRNA #3 (S3) proving most effective in silencing FOSL1 and subsequently chosen for further investigations. Subsequently, we assessed the impact of FOSL1 knockdown on ox‐LDL‐induced inflammatory response in THP1‐derived macrophages. Figure 6e illustrates that silencing FOSL1 partially suppresses the expression of NLRP3 and pP65 induced by ox‐LDL, which are regulators of inflammation mediators. Furthermore, downregulation of FOSL1 suppresses the ox‐LDL‐induced expression of IL‐6, IL‐8, and IL‐1*β* (Figure 6f,h) while increasing IL‐10 levels (Figure 6i). These findings suggest that FOSL1 promotes the ox‐LDL‐induced inflammatory response in THP1‐derived macrophages.

Figure 6Silencing FOSL1 suppressed ox‐LDL‐induced THP1‐derived macrophage inflammatory response. (a, b) The level of FOSL1 mRNA and protein was evaluated by qPCR and Western blot after THP1‐derived macrophage were incubated with indicated doses ox‐LDL for 24 h. THP1 cells were transfected with NC and three FOSL1 siRNA oligos for 24 h, respectively. (c, d) qPCR and Western blot detected the expression of FOSL1 mRNA and protein. THP1 cells were transfected with LncRNA CCAT1 siRNA for 24 h, followed by a 24‐h incubation with 100 ng/mL of PMA to induce THP1‐derived macrophage. Subsequently, the cells were treated with 50 *μ*g/mL of ox‐LDL for an additional 24 h. (e) Western blot detected the expression of NLRP3 and pP65 protein. (f–i) ELISA method evaluated the levels of IL‐8, IL‐6, IL‐1*β*, and IL‐10 in culture medium. ^ns^ *p* ≥ 0.05 and  ^∗^ *p* < 0.05 by one‐way ANOVA followed by Tukey′s multiple comparisons test. Experiment was conducted with three replicates.

**Figure:**
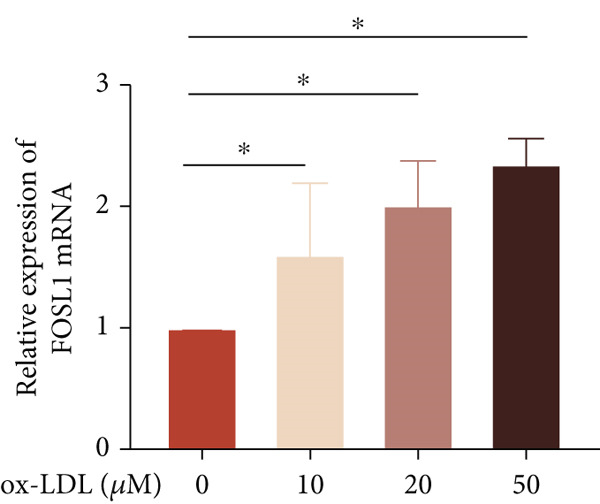
(a)

**Figure:**
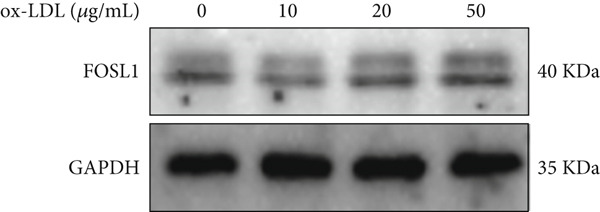
(b)

**Figure:**
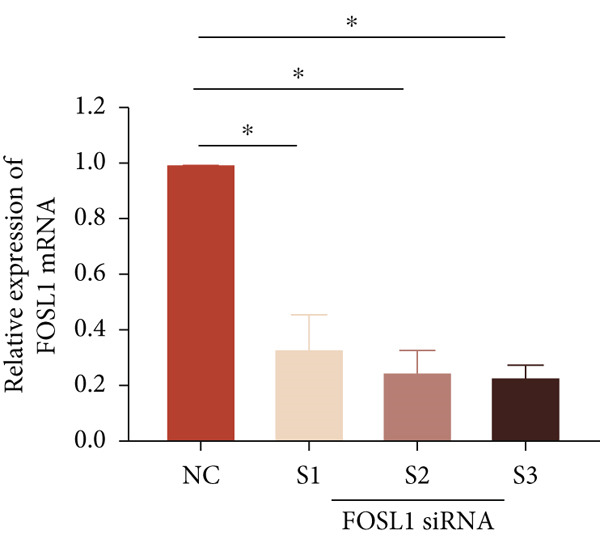
(c)

**Figure:**
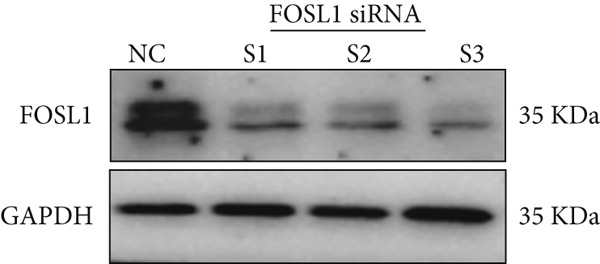
(d)

**Figure:**
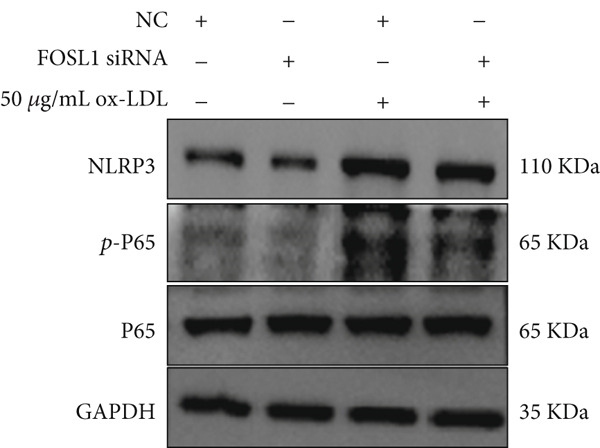
(e)

**Figure:**
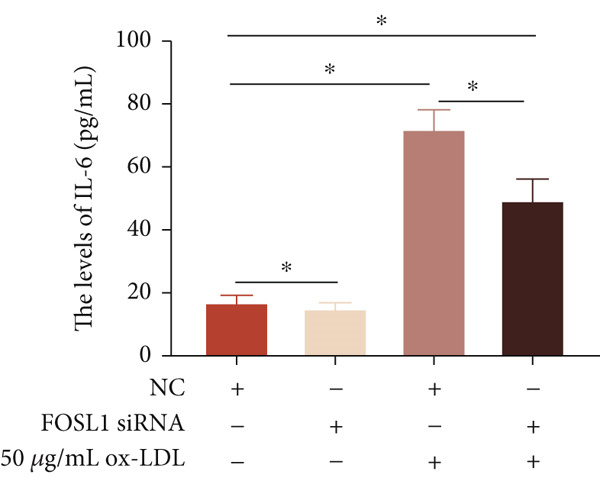
(f)

**Figure:**
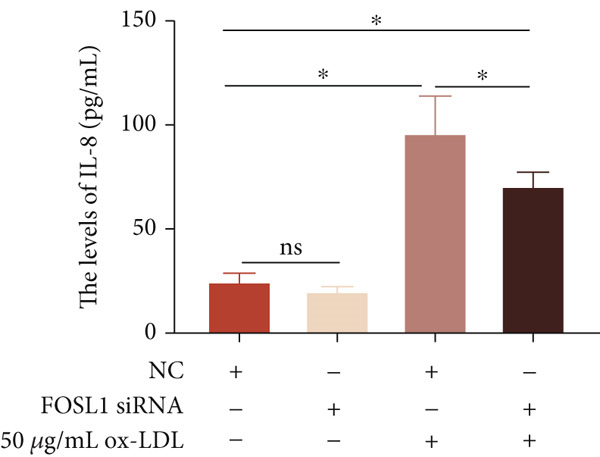
(g)

**Figure:**
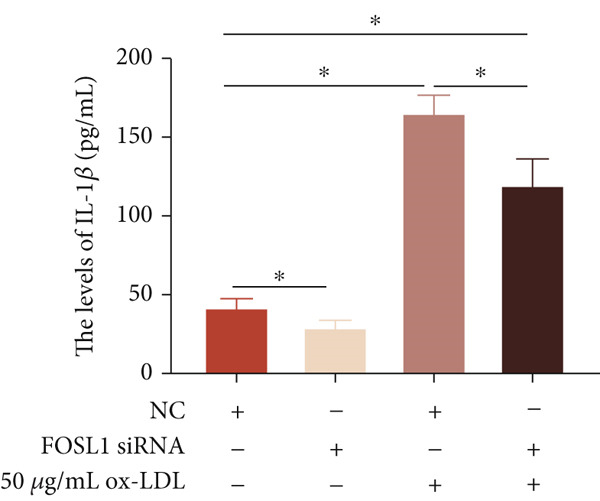
(h)

**Figure:**
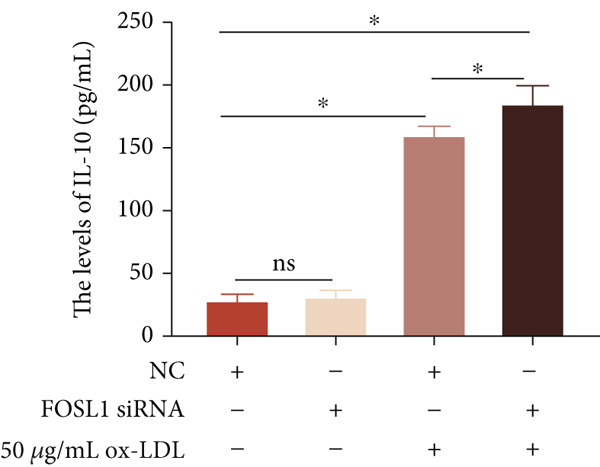
(i)

## 4. Discussion

In this study, we investigated the role and underlying mechanism of LncRNA CCAT1 in THP1‐derived macrophages exposed to ox‐LDL. The principal findings were twofold: (i) ox‐LDL significantly upregulated the expression of LncRNA CCAT1 in macrophages, whereas LncRNA CCAT1 silencing markedly attenuated the ox‐LDL‐induced inflammatory response and (ii) this elevated LncRNA CCAT1 sponged miR‐296‐3p to consequently derepress its target, FOSL1, thereby amplifying the inflammatory cascade in ox‐LDL‐stimulated macrophages.

Increasing evidence suggests that a thorough understanding of the roles and underlying mechanisms of LncRNAs in the ox‐LDL‐induced inflammatory response in macrophages may present a viable target for AS therapy [20–23]. Nevertheless, our current knowledge of the pathophysiological role of LncRNA CCAT1 in this condition remains limited. This study demonstrates that ox‐LDL‐induced elevated levels of LncRNA CCAT1 in THP1‐derived macrophages. Suppressing LncRNA CCAT1 expression alleviated the inflammatory response, consistent with previous findings [18, 24]. These findings indicate that LncRNA CCAT1 plays a pivotal role in macrophage inflammation and potentially contributes to the progression of AS.

LncRNA sponging of miRNAs is a primary method for mediating downstream gene expression by alleviating the inhibitory effect of miRNAs on target mRNA. Various studies have demonstrated that LncRNA CCAT1 acts as a sponge for miRNAs such as let‐7, miR‐218‐5p, and miR‐490‐3p to enhance the downstream expression of cMyc, MTF2, and TGF*β*R1 [25–27]. Here, we identified that LncRNA CCAT1 sponges miR‐296‐3p in THP1‐derived macrophages. Given the crucial role of miRNAs in AS development, particularly in modulating macrophages′ inflammatory responses, recent studies have emphasized their significant regulatory influence [28]. Our research further supports that miR‐296‐3p exerts an anti‐inflammatory effect by targeting the FOSL1 gene, consistent with previous findings [29]. These results suggest that the sponging of miR‐296‐3p by LncRNA CCAT1 could represent a critical mechanism of ox‐LDL‐induced inflammatory response, potentially influencing AS progression.

FOSL1, also known as Fra‐1, is a member of the FOS family of transcription factors and a key component of the activator protein 1 (AP‐1) complex [30, 31]. Extensive research has implicated FOSL1 as a critical regulator in various pathologies, particularly in cancer, where it governs tumorigenicity, proliferation, and metastasis [2, 32–34]. Crucially, its role extends to inflammatory diseases. For instance, FOSL1 is highly expressed in vascular atherosclerotic lesions compared to healthy tissue [35], and its suppression has been shown to reduce the production of ox‐LDL‐induced inflammatory mediators [36]. Mechanistically, the pro‐inflammatory function of FOSL1 is well‐documented. Studies have demonstrated that FOSL1 knockout in alveolar macrophages attenuates the expression of IL‐1*β* and macrophage inflammatory protein‐1*α* while increasing anti‐inflammatory IL‐10 [37]. Others have found that silencing FOSL1 inhibits NLRP3 inflammasome and NF‐*κ*B p65 activation [38]. Furthermore, in an arthritis model, LPS‐induced FOSL1 was shown to directly bind to the *Arginase 1* (*Arg1*) promoter, inhibiting its transcription and promoting macrophage polarization toward the proinflammatory M1 phenotype [39]. Similarly, our study found that FOSL1 expression was significantly elevated in THP1‐derived macrophages following ox‐LDL exposure. Moreover, we demonstrated that FOSL1 downregulation suppressed the expression of both NLRP3 and phosphorylated p65 (p‐p65). Given the established roles of the NLRP3 inflammasome and NF‐*κ*B signaling in driving inflammatory mediator production [40, 41], our findings suggest that FOSL1 amplifies the ox‐LDL‐induced inflammatory response in macrophages, likely by reinforcing their polarization toward an M1‐like state.

This study had several limitations. Firstly, the levels of LncRNA CCAT1 should be assessed in the atherosclerotic plaque and monocytes from peripheral blood of atherosclerotic patients, while investigating the correlation between LncRNA CCAT1 levels and AS progression. Secondly, the inhibitory effect of silencing LncRNA CCAT1 on the inflammatory response and its impact on the progression of AS should be validated in an *in vivo* model. Utilizing macrophage‐specific LncRNA CCAT1 knockout mice may offer more specific evidence to elucidate the role of LncRNA CCAT1 in AS. Thirdly, further studies are needed to investigate the novel mechanism by which FOSL1 regulates the inflammatory response induced by ox‐LDL.

In conclusion, our study illustrates that LncRNA CCAT1 enhances the inflammatory response triggered by ox‐LDL in THP1‐derived macrophages by acting as a miR‐296‐3p sponge and elevating FOSL1 levels. These results imply that targeting LncRNA CCAT1 for silence could offer a promising therapeutic strategy in the prevention and treatment of atherosclerotic CVDs.

## Disclosure

All authors reviewed and approved the final manuscript.

## Conflicts of Interest

The authors declare no conflicts of interest.

## Author Contributions

J.Y. and B.H. designed and supervised the study. H.Z., F.L., and B.H. were responsible for data collection, analysis, and manuscript writing, while G.M., Y.W., L.Z., C.Y., K.H., F.G., J.A., and B.H. were responsible for data collection and analysis. H.Z., J.Y.. and B.H. contributed to preparing figures and tables. J.Y. and B.H. were responsible for conducting the manipulation. H.Z. and F.L. have contributed equally to this work.

## Funding

This funding was supported by the Kunming Health Science and Technology Personnel Training Project Medical Technology Center Construction Project, 2022‐SW(Technology)‐34.

## Supporting information


**Supporting Information** Additional supporting information can be found online in the Supporting Information section. The supporting information includes the details of the primer sequences of the target gene.

## Data Availability

Data is available on request from the authors.
