# Supplementary material for: Knockdown of LncRNA CCAT1 Attenuates ox‐LDL‐Induced Inflammation in THP1‐Derived Macrophages via the miR‐296‐3p/FOSL1 Axis
Source: Cardiovasc Ther. 2025 Dec 22;2025:9277233. doi: 10.1155/cdr/9277233 (PMC12723178; doi:10.1155/cdr/9277233)
Supplement: Supplementary file 1 — Supporting Information Additional supporting information can be found online in the Supporting Information section. The supporting information includes the details of the primer sequences of the target gene. [file CDR-2025-9277233-s001.zip › 2025-9-11-supplement file 1.docx]

**LncRNA CCAT1 knockdown inhibits ox-LDL-induced inflammatory Response in THP1 derived-macrophages through the miR-296-3p/ FOSL1 axis**

Haiping Zhang^1^†, Feng Liu^1,2^†, Guihua Miao^1^, Yijiao Wang^1^, Lijun Zhang^1^, Chun Yang^1^, Kai Huang^1^, Fengyun Guo^1^, JunBo An^1^, Binfeng He^3*^, Jinshan Ye^1*^

^1^Department of Cardiology, Tongren Hospital, Yunnan 650032, China

^2^Department of Cardiology, Jintang County First People's Hospital, Sichuan 610400, China

^3^Department of General Practice, Xinqiao hospital, Chongqing, 400037, China

*Correspondence

Binfeng He, Email: [ldhbf@tmmu.edu.cn](mailto:ldhbf@tmmu.edu.cn)

Jinshan Ye, Email: [doctoryjs@163.com](mailto:doctoryjs@163.com)

†These authors contributed equally to this work

**Supplementary Table 1**

| Name | Sequence |
| --- | --- |
| CCAT1-F | 5’-CACCTACGCATACCTCTGCTTC-3’ |
| CCAT1-R | 5’-TGATTGCTCCTGTTTCCCTTTG-3’ |
| FOSL1-F | 5’-CAGGCGGAGACTGACAAACTG-3’ |
| FOSL1-R | 5’-TCCTTCCGGGATTTTGCAGAT-3’ |
| GAPDH-F | 5’-GGAGCGAGATCCCTCCAAAAT-3’ |
| GAPDH-R | 5’-GGCTGTTGTCATACTTCTCATGG-3’ |
| miR-296-3p-F | ATTATAGAGGGTTGGGTGGAGGCT |
| let-7a-F | CCTGAGGTAGTAGGTTGTGTGGTT |
| miR-454-3p-F | CCGTAGTGCAATATTGCTTATAGGGT |
| miR-152-3P-F | CGTCAGTGCATGACAGAACTTGG |

*:Universal PCR Primer R and Universal U6 Primer F is provided from miRNA First Strand cDNA Synthesis Kit (Tailing Reaction).
