# Supplementary material for: Knockdown of LncRNA CCAT1 Attenuates ox‐LDL‐Induced Inflammation in THP1‐Derived Macrophages via the miR‐296‐3p/FOSL1 Axis
Source: Cardiovasc Ther. 2025 Dec 22;2025:9277233. doi: 10.1155/cdr/9277233 (PMC12723178; doi:10.1155/cdr/9277233)
Supplement: Supplementary file 1 — Supporting Information Additional supporting information can be found online in the Supporting Information section. The supporting information includes the details of the primer sequences of the target gene. [file CDR-2025-9277233-s001.zip › 2025-9-11-supplement file 2.docx.pdf]

LncRNA	MiRNA	Pubmed	Source
CCAT1	miR-181b	28475287	LncACTdb
CCAT1	miR-33a	28409554	LncACTdb
CCAT1	let-7a	25884472	LncACTdb
CCAT1	let-7b	25884472	LncACTdb
CCAT1	let-7c	25884472	LncACTdb
CCAT1	let-7e	25884472	LncACTdb
CCAT1	miR-1290	29424889	LncACTdb
CCAT1	miR-181a-5p	29228867	LncACTdb
CCAT1	miR-7	27956498	LncACTdb
CCAT1	miR-218-5p	28088735	LncACTdb
CCAT1	miR-410	29190961	LncACTdb
CCAT1	miR-490-3p	28381168	LncACTdb
CCAT1	miR-218	28631575	LncACTdb
CCAT1	miR-148a	28549102	LncACTdb
CCAT1	miR-148b	29024383	LncACTdb

LncRNA	MiRNA	Pubmed	Source
CCAT1	let-7	27830017	LncACTdb
CCAT1	miR-155	26923190	LncACTdb
CCAT1	miR-490	26825578	LncACTdb
CCAT1	miR-218-5p	25569100	LncACTdb
CCAT1	hsa-miR-543	-	Starbase
CCAT1	hsa-miR-148a-3p	-	Starbase
CCAT1	hsa-miR-4295	-	Starbase
CCAT1	hsa-miR-181d-5p	-	Starbase
CCAT1	hsa-miR-181c-5p	-	Starbase
CCAT1	hsa-miR-24-3p	-	Starbase
CCAT1	hsa-miR-454-3p	-	Starbase
CCAT1	hsa-miR-152-3p	-	Starbase
CCAT1	hsa-miR-301b	-	Starbase
CCAT1	hsa-miR-3666	-	Starbase
CCAT1	hsa-miR-148b-3p	-	Starbase

LncRNA	MiRNA	Pubmed	Source
CCAT1	hsa-miR-130a-3p	-	Starbase
CCAT1	hsa-miR-410-3p	-	Starbase
CCAT1	hsa-miR-181b-5p	-	Starbase
CCAT1	hsa-miR-216a-5p	-	Starbase
CCAT1	hsa-miR-218-5p	-	Starbase
CCAT1	hsa-miR-181a-5p	-	Starbase
CCAT1	hsa-miR-4262	-	Starbase
CCAT1	hsa-miR-490-3p	-	Starbase
CCAT1	hsa-miR-130b-3p	-	Starbase
CCAT1	hsa-miR-301a-3p	-	Starbase
CCAT1	hsa-miR-296-3p	-	Starbase
